# Dietary Nitrate Is a Modifier of Vascular Gene Expression in Old Male Mice

**DOI:** 10.1155/2015/658264

**Published:** 2015-03-08

**Authors:** Christos Rammos, Matthias Totzeck, René Deenen, Karl Köhrer, Malte Kelm, Tienush Rassaf, Ulrike B. Hendgen-Cotta

**Affiliations:** ^1^Division of Cardiology, Pulmonology, and Vascular Medicine, Medical Faculty, University Hospital Düsseldorf, Moorenstraße 5, 40225 Düsseldorf, Germany; ^2^Biological and Medical Research Center (BMFZ), Genomics and Transcriptomics Laboratory (GTL), Heinrich-Heine-University, Universitätsstraße 1, 40225 Düsseldorf, Germany; ^3^Cardiovascular Research Institute Düsseldorf (CARID), Medical Faculty, University Düsseldorf, Moorenstrasse 5, 40225 Düsseldorf, Germany

## Abstract

Aging leads to a number of disadvantageous changes in the cardiovascular system. Deterioration of vascular homoeostasis with increase in oxidative stress, chronic low-grade inflammation, and impaired nitric oxide bioavailability results in endothelial dysfunction, increased vascular stiffness, and compromised arterial-ventricular interactions. A chronic dietary supplementation with the micronutrient nitrate has been demonstrated to improve vascular function. Healthy dietary patterns may regulate gene expression profiles. However, the mechanisms are incompletely understood. The changes that occur at the gene expression level and transcriptional profile following a nutritional modification with nitrate have not been elucidated. To determine the changes of the vascular transcriptome, we conducted gene expression microarray experiments on aortas of old mice, which were treated with dietary nitrate. Our results highlight differentially expressed genes overrepresented in gene ontology categories. Molecular interaction and reaction pathways involved in the calcium-signaling pathway and the detoxification system were identified. Our results provide novel insight to an altered gene-expression profile in old mice following nitrate supplementation. This supports the general notion of nutritional approaches to modulate age-related changes of vascular functions and its detrimental consequences.

## 1. Introduction

Aging is considered to be the most important nonmodifiable cardiovascular risk factor, leading to increased cardiovascular morbidity and mortality [1]. It deteriorates vascular integrity and entails a number of disadvantageous structural and functional changes on the arterial system [2]. Structural alterations include increased luminal diameter, vascular wall remodeling with thickened intima and media, and altered extracellular matrix content [2]. Functional changes are characterized by increased aortic stiffness, endothelial dysfunction, and subsequent systolic hypertension. These alterations are regulated by signaling pathways activated by endogenous and exogenous factors [3–5]. On a cellular level, decreased protein synthesis, dysfunctional mitochondria, oxidative stress, impaired antioxidant defense mechanisms, disturbed calcium handling, and increased DNA and protein oxidation have been proposed [6–9]. On the genomic level, we recently identified differences in the transcriptional profile and the genes potentially responsible for the vascular aging process, with emphasis on the detoxification system [10].

Dietary interventions have emerged as novel options, counteracting age-related vascular dysfunction [11–14]. A definite composition of healthy diet is still elusive. Nevertheless, it is known, particularly since the DASH (dietary approaches to stop hypertension) trial, that certain dietary patterns, for example, the so-called Mediterranean diet, positively influence blood pressures and cardiovascular outcome [15–18].

Inorganic nitrate is abundant in our everyday diet and especially in leafy greens. The nutritional aspects of nitrate are intriguing since it represents a source for the endogenous generation of nitrite, nitric oxide (NO), and other reactive nitrogen oxides [19–21]. In experimental studies and first clinical observations, dietary inorganic nitrate interventions have emerged as novel nutrient options modulating cardiovascular functions. This relates to regenerative processes in ischemic tissues, enhancement of endothelial function, and limitation of mitochondrial reactive oxygen species in a cardiomyopathy model [22–24]. We have demonstrated that a chronic nitrate supplementation improves vascular stiffness and reduces systolic blood pressures and the level of proinflammatory cytokines in elderly volunteers [25, 26]. However, to comprehend the beneficial effects of a nitrate rich diet on the vasculature the underlying cellular and molecular changes have to be characterized.

Nitrate-derived nitrite and NO exert beneficial effects through second messengers, posttranslational modifications, and regulation of gene expression [27–29]. The genes potentially affected by a nitrate rich diet counteracting the vascular aging process have not been identified so far. We therefore set out to compare the transcriptional profile in aged aortas of old nitrate-treated mice with age-related endothelial dysfunction applying a high-throughput genomic screening approach. Using microarray analyses, identification of genes, and expression differences was simultaneously accomplished. Our study discloses alterations in gene expression following a dietary nitrate supplementation in the aged vascular system.

## 2. Material and Methods

### 2.1. Animals

Male C57BL/6 mice were obtained from Janvier (Saint Berthevin, France) and kept one week in the local animal house for acclimatization. Old (20 months) mice were housed in controlled temperature and light conditions with feeding (V1534-000, Sniff, Soest, Germany) and watering* ad libitum*. A group of old mice received 1 g/L sodium-nitrate (NaNO_3_, ~150 *μ*moles according to the measured daily drinking water consumption) in their drinking water for 8 weeks, as described previously [23, 26]. An equal concentration of sodium chloride served as control. For aortic vasoreactivity studies young (6 months) mice served as controls. For the gene-expression experiments different mice from the same supplier were used. The care and all experiments were performed according to the animal welfare regulations of the German local authorities conforming to NIH Guidelines.

### 2.2. Aortic Vascular Reactivity

*Ex vivo* endothelial functions were assessed using an aortic ring bioassay, as described [28, 30]. Briefly, three 2-3-mm wide aortic rings were prepared from each mouse, perivascular fat was removed and aortas were suspended in an organ bath containing 10 mL modified Krebs-Henseleit buffer and connected to an isometric force transducer (EMKA Technologies, Paris, France). Organ chambers were aerated continuously with 95% O_2_ and 5% CO_2_ to achieve pH of 7.4. Isometric tension was recorded continuously. The vascular rings with intact endothelium and similar dimensions equilibrated to a resting tension of 1.0 g. All rings were preconstricted with KCl (40 mM) and, after washout, phenylephrine (10 *μ*M). Endothelium function was measured as the relaxation response to acetylcholine (from 10^−9^ to 10^−4^ M). Endothelium-independent relaxation was determined as the response to sodium nitroprusside (from 10^−9^ to 10^−2 ^M).

### 2.3. Chemicals

All reagents were obtained from Sigma-Aldrich (Taufkirchen, Germany) unless indicated otherwise.

### 2.4. RNA Preparation and Microarray Assays

RNA preparation and microarray assays were conducted as described previously [10]. Briefly, the descending aorta of each mouse was used. The vessel was flushed thoroughly with ice-cold PBS, through the left ventricle of the heart, cleaned of periadventitial fat and connective tissues, snap-frozen in liquid nitrogen, and stored at −80°C. Extraction of total RNA from the tissue was performed (Qiagen RNeasy Mini Kit, Qiagen, Hilden, Germany). Removal of residual genomic DNA was achieved by treatment with DNase I. Total RNA preparations were checked for RNA integrity (Agilent 2100 Bioanalyzer, Agilent Technologies, Palo Alto, CA). All samples showed common high quality RNA Integrity Numbers (RIN 9.0–9.6) and RNA was quantified by photometric Nanodrop measurement.

Synthesis of cDNA and subsequent fluorescent labeling of cRNA was done on six replicates of each group (old and old nitrate treated mice) according to the manufacturers protocol (One-Color Microarray-Based Gene Expression Analysis/Low Input Quick Amp Labeling; Agilent Technologies, Palo Alto, CA). Briefly, 100 ng of total RNA was converted to cDNA, followed by* in vitro* transcription and incorporation of Cy3-CTP into nascent cRNA. After fragmentation labeled cRNA was hybridized to Agilent SurePrint G3 Mouse GE 8x60K Microarrays for 17 h at 65°C. Quality control parameters of cRNA labeling and hybridization performance were found within the manufacturers specifications. Arrays were scanned as described by the manufacturer. Signal intensities on 20 bit tiff images were calculated by Feature Extraction software (FE, Vers. 10.7.1.1; Agilent Technologies, Palo Alto, CA).

GeneSpring GX software was used to analyze data (Vers. 12.5; Agilent Technologies, Palo Alto, CA). Probe signal intensities were quantile normalized across all samples to reduce interarray variability [31]. Input data preprocessing was concluded by baseline transformation to the median of all samples. Biological replicates were grouped according to their respective experimental condition. A given transcript had to be expressed above background (“detected” by GeneSpring GX) in at least five of six replicates in any one of two, or both conditions to be additional analyzed in pairwise comparisons of conditions. Hierarchical clustering of gene expression patterns identified no obvious outlier samples. Differential gene expression was determined statistically by a moderated *t*-test with genes showing *P* < 0.05 considered to be significantly differentially expressed [32].

The microarray data set as been deposited in the NCBI Gene Expression Omnibus (GEO) public database in compliance with MIAME (Minimum Information About a Microarray Experiment) guidelines (GEO series accession number GSE66572).

### 2.5. Functional Analysis by Gene Ontology and Gene Pathways

GeneSpring GX and Database for Annotation, Visualization, and Integrated Discovery (DAVID Bioinformatics database) was used to identify functional related categories of genes.

Differentially expressed genes (*P* < 0.05) with an additional fold change > 1.2 were considered relevant and were imported into DAVID Bioinformatics database [33, 34]. The DAVID software analysed significant enrichment of differentially expressed genes within gene ontology (GO) terms and additionally involves assessment of advanced pathway analysis [35, 36]. The background was chosen from the probes represented on the Agilent 8x60K mouse array, which were detected on either one of the three conditions to determine nitrate-associated genes in the aortic tissue. A modified Fisher's exact test assessed the significance of the association between the observed data and the data in the GO and canonical pathway. Each identified pathway was assigned a significance score, defined by the number of differentially regulated focus genes in the data set. This score was the negative logarithm of the *P* value, indicative of the likelihood that genes are found in a pathway randomly.

### 2.6. Statistical Analysis

The results of endothelial functions are presented as mean ± SEM. Data were analyzed by 1-way ANOVA and posthoc Bonferroni multiple comparison correction (for all pairwise tests) with GraphPad Prism 5 (GraphPad, California) software to compare differences between groups. A *P* value of <0.05 was considered significant. Agilent microarray probe sets were analyzed as described above.

## 3. Results and Discussion

### 3.1. Dietary Nitrate Reverses Age-Related Endothelial Dysfunction

**Figure 1:**
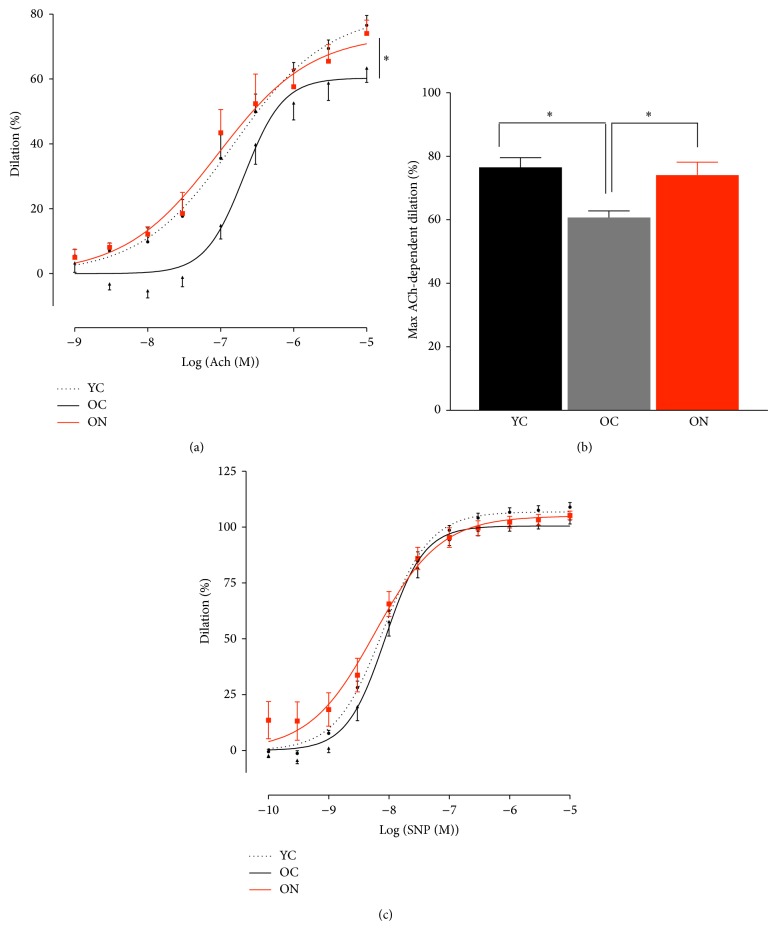
Age-related endothelial dysfunction is reversed by dietary nitrate supplementation. (a) and (b) endothelium dependent function determined by dose-responses and maximum vasodilation to the endothelium-dependent dilator acetylcholine (ACh) in young, old, and nitrite-supplemented old male mice (YC, OC and ON). (c) Dose-responses to the endothelium-independent vasodilator sodium nitroprusside (SNP). (Values are mean ± SEM, *n* = 6, ^*^ *P* < 0.05.)

We studied old C57BL/6 mice and characterized age-related endothelial functions and the reversal through dietary nitrate. Using our model of aged mice, we were able to demonstrate an age-related vascular phenotype and associated alterations in vascular gene expression pattern [10]. Endothelial dysfunction is an important change that occurs in aged vessels, within increased cardiovascular risk and declining with advancing age [37, 38]. Decreases in endothelium-dependent dilator responses are believed to be a result of alterations in NO bioavailability, activity of the endothelial NO synthase, and increased formation of reactive oxygen species (ROS) and gene expression. We here show that aged mice have attenuated endothelial function as determined in isolated aortic bioassay studies compared to young mice (max vasodilator response to acetylcholine (ACh) young 76.6 ± 3.0% versus old 60.7 ± 2.1%, *P* = 0.003, Figures 1(a) and 1(b)). Importantly, following a chronic dietary nitrate supplementation a reversal of endothelial dysfunction was observed (max. vasodilator response to ACh old nitrate 60.7 ± 2.1% versus 74.1 ± 4.1%, *P* = 0.018, Figures 1(a) and 1(b)). No effect was observed for endothelium-independent function in young, old and old nitrate treated mice (Figure 1(c)). Our results are in line with previous findings showing decreased age-related endothelial functions and improvements through nitrate supplementation in elderly humans [25]. We have previously shown that a chronic nitrate supplementation regimen affects plasma nitrite and nitrate levels [23]. Although this dosage is relatively high as compared to what can be ingested from a diet, it yields the equivalent effect a diet rich in vegetables exerts in humans [25, 26]. In mice, a diet rich in nitrite led to a decrease in inflammatory cytokines, suggesting an enhancement in NO bioavailability through inhibition of inflammation and inactivation of ROS [11].

### 3.2. Microarrays, Gene Ontology, and Pathway Analysis

Dietary interventions are known to influence cardiovascular functions and impact gene expression patterns [39–42]. To explore the genes that underlie and are responsible for the beneficial effects of nitrate supplementation in aged mice we performed gene expression microarray experiments on whole thoracic aortas of old and old nitrate treated mice. Microarrays allow the simultaneous quantification of gene expression differences, obtaining insights in health and disease [43]. Aging as well as dietary interventions lead to subtle changes of the transcriptional profile and accordingly, we choose a statistical significance in differential gene expression with a *P*-value set to <0.05, not performing control for false discovery rate or family-wise error rate, as previously reported [10, 40, 44, 45].

**Figure 2:**
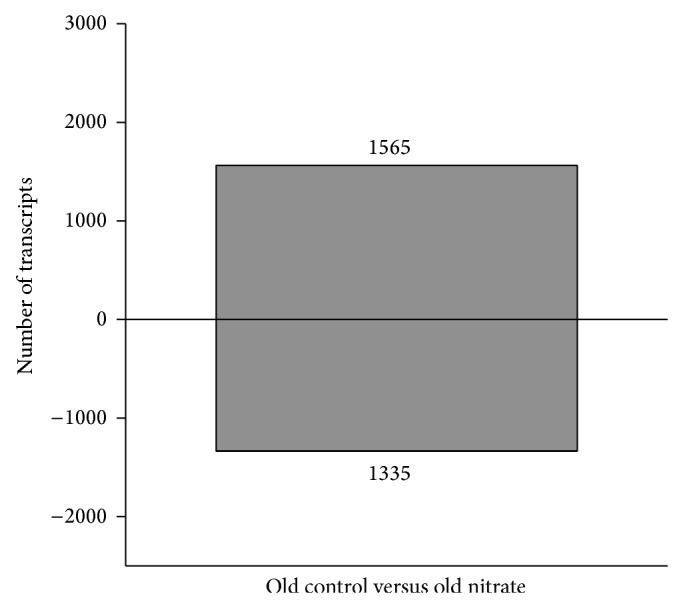
Differentially expressed transcripts in old male C57BL/6 aortas following nitrate treatment (*n* = 6; determined from total 26,774 transcripts; fold change > 1.2; *P* < 0.05). Old control versus old nitrate yielded 1565 upregulated and 1335 downregulated transcripts.

Overall probe sets on the chip before filtering were 55682 and after exclusion of undetectable transcripts 26621 expressed transcripts were found. About 2900 transcripts were expressed differentially in aortic tissue of old and old nitrate treated mice, with totally 1565 upregulated and 1335 downregulated transcripts (Figure 2), which correspond to 2231 genes. Since the absolute numbers in gene expression differences do not provide explanations and comprehension of changes that occur through a dietary modification in aged mice, we performed GO and pathway analysis to gain insight into the transcriptional changes following nitrate supplementation.

Pathway analysis was performed through DAVID Bioinformatics database to identify the biological processes associated to differential regulation in old nitrate treated mice. DAVIDs functional annotations tool identifies enriched GO annotations categorized in biological processes, molecular functions and cellular components, with highest GO terms displayed in Table 1.

**Figure 3:**
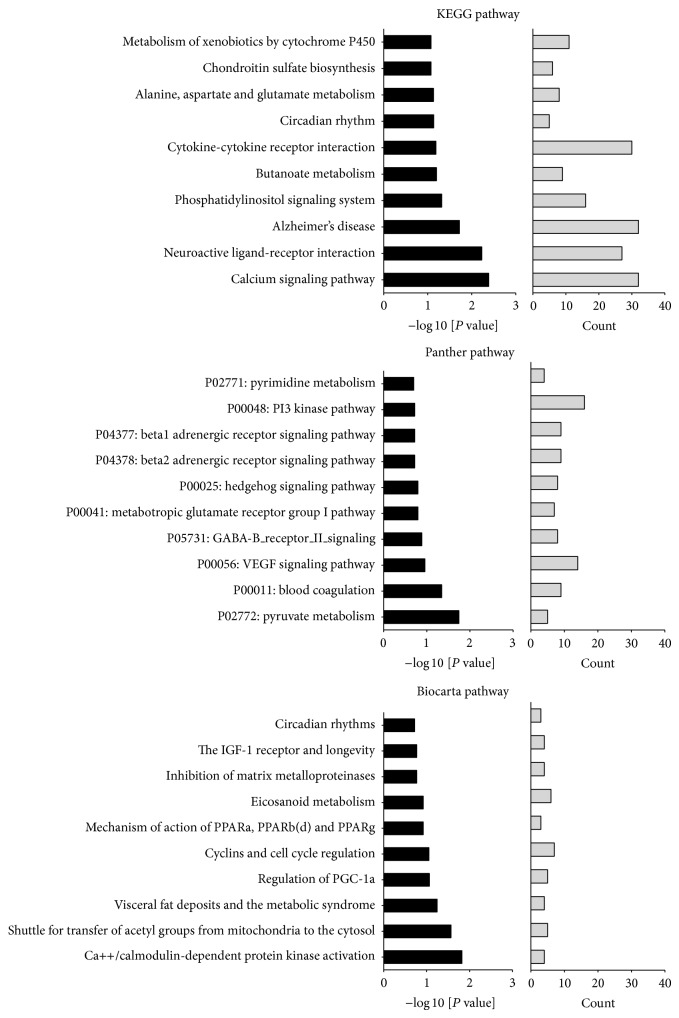
Enriched pathways in aortas of old nitrate treated male mice. Highest enriched KEGG, Panther, and Biocarta pathways are displayed of old aortic tissue following nitrate supplementation. Individual pathways and corresponding gene count are sorted by negative log [*P* value].

To determine the targets altered by a dietary nitrate supplementation in old mice we conducted DAVID's pathway analysis [33, 46]. The highest enriched databases of pathways that were in our study were KEGG, Panther, and Biocarta.  Figure 3 depicts overrepresented pathways in aortas of old nitrate treated mice with the corresponding gene count. One of the highest enriched pathways was “calcium signaling” in the KEGG database and the “calcium/calmodulin dependent protein kinase activation” in Biocarta (Figure 3).

The calcium signaling pathways were followed by pathways related to neurological and brain function “neuroactive ligand-receptor interaction,” “Alzheimer disease,” and “GABA-B receptor II signaling.” This might be of particular interest given a potentially impaired NO bioavailability in neurological diseases like Alzheimer's disease [47, 48]. The possible link gained from our study is of importance since dietary nitrate was shown to improve regional brain perfusion in older adults and to improve reaction time in diabetic patients [49, 50]. NO signaling was additionally linked to neuroprotective signaling pathways and to promote survival and inhibit apoptotic cell death in neuronal cell types [51]. On the contrary, a recent study showed no effects on cognitive performance in older adults after short-term beetroot juice ingestion [52]. However, since we used a chronic supplementation regimen in mice a direct conclusion remains unfeasible.

### 3.3. Calcium Signaling Pathway

**Figure 4:**
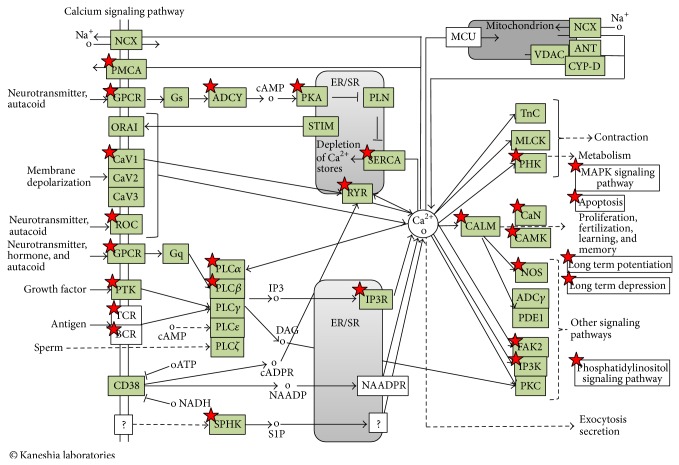
Calcium signaling pathway in old nitrate treated aorta. KEGG-pathway analysis determined by DAVID Bioinformatics resources showing differentially expressed genes highlighted with red stars (for up-/downregulation refer to Table 2).

Aging leads to alterations of vascular gene expression which ultimately lead to increased vascular tone perpetuating arterial stiffness and hypertension. Increased age-related stiffness has been demonstrated to be an independent predictor of CVD events [53]. Comparing old and old nitrate treated mice we found KEGGs “calcium signaling” and Biocartas “calcium/calmodulin dependent protein kinase activation” to be the highest enriched pathways in old mice following nitrate supplementation. Calcium signaling is fundamental to vascular functions and the vascular tone is maintained by a highly regulated calcium homeostasis. In smooth muscle cells calcium is the main trigger for the action potential, calcium-dependent phosphorylation of the myosin light chain leads to an increase in muscle cell tone and the changes in intracellular calcium parallel the changes in contractile forces. We and others have recently shown that a dietary nitrate supplementation reduces arterial stiffness and blood pressure in older adults and healthy volunteers [25, 54, 55]. While acute effects are exerted by nitrate to nitrite bioconversion and subsequent reduction to NO, the long-term effects are incompletely understood. NO is known to modulate calcium signaling in the cardiovascular system, regulating important calcium handling proteins, for example, SERCA (sarcoplamatic reticulum calcium pump), ryanodine receptor, and L-Type calcium hannel, which are responsible for smooth muscle cell relaxation [56–58]. Recently, it was shown that ingestion of nitrate over 7 days increased sarcoplasmatic reticulum calcium release and tetanic contractile force production via alterations in cellular calcium handling with altered calcium handling protein expressions in nitrate treated mice [59]. We now identified *n* = 30 differentially expressed genes related to calcium pathway in old versus old nitrate treated mice (Table 2 and Figure 4). Major players of the cellular calcium handling process were found, namely the L-type calcium channel (Cacna1d and Ppapdc2), the sarcoplasmatic calcium release channel (Ryanodine Receptor 2, Ryr2), the calcium/calmodulin-dependent protein kinase type II (Calm2, Camk2g, Camk4), and the inositol triphosphate receptor (Itpr2, Itpr3, Itpka; for detailed regulation and fold change expression difference refer to Table 2).

### 3.4. Detoxification, Antioxidation, and Free Radical Removal

The vascular aging process is characterized by increased oxidative stress and impaired antioxidant defense mechanisms [6, 60]. We have recently shown a distinctive transcriptomic profile with altered expression patterns in the old aorta with emphasis on the detoxification system [10]. Oxidative stress progresses due to excessive generation of ROS, to reduced antioxidant capacity or due to uncoupled nitric oxide synthase, amongst others. We have demonstrated that nitrite-derived NO impacts ROS generation and additionally reduces protein damage in myocardial ischemia/reperfusion injury [30, 61]. It was evidenced that NO regulates cardiovascular cell signaling and modulates cellular energetics [62, 63]. While in the acute setting nitrite is reduced by nitrite reductases and exerts vascular effects, the consequences of a long-term administration are unclear [64–66]. Recently, decreased inflammatory cytokines, inhibition of inflammation and inactivation of ROS following a nitrite rich diet in mice and healthy volunteers with subsequent improved age-related vascular functions were observed [11, 26, 67]. We now determined the enriched genes in aortas of old nitrate treated mice associated with the biological process of detoxification, antioxidation, and free radical removal as defined by Panther GO terms (Table 3). Most of the genes altered by nitrate in the old aorta were related to glutathione metabolism, the major antioxidant system. Remarkably, a downregulation of inducible nitric oxide synthase was noted, which could suggest a reduced inflammation in nitrate treated animals. Of note, the diversity of transcriptional regulation is observed through the bilateral regulation of superoxide dismutases (Table 3).

### 3.5. Limitations

We used the whole thoracic aorta instead of a single cell type. Although a specific cell might provide fundamental information, cell-cell interactions are imperative for coordinated organ functions and we thus intended to examine the complete transcriptional vascular changes that occur following nitrate supplementation. As we used only male mice our results might not apply to female mice and this should be taken into account for further studies. Moreover, alternative splicing variants are inadequately reflected by our analysis. Verification of microarray data is needed in forthcoming studies to confirm the beneficial effects of dietary nitrate on vascular tissue. Important targets to confirm would be the family of superoxide dismutases (SOD 1 and 3) the nitric oxide synthase 2, the platelet-derived growth factor beta (PDGFR-beta), and the adrenergic receptor. Also, measurement of markers of oxidative stress or inflammation would be helpful to verify the presented findings. Finally, it has to be noted that gene expression data does not always correspond to protein expression and activity.

## 4. Conclusions

Aging is the most important nonmodifiable cardiovascular risk factor, which leads to disadvantageous changes of the vasculature. A dietary intervention with inorganic nitrate has been evidenced to improve vascular functions. We determined changes that occur at a gene expression level and transcriptional profile following a chronic nitrate supplementation in aged mice. Our results highlight differentially expressed genes overrepresented in GO categories, pathways related to the calcium-signaling, the detoxification and antioxidation system. Our results contribute to existing knowledge of nitrate's beneficial effects. This supports the concept of nutritional approaches to modulate age-related changes of vascular functions and its detrimental consequences.

## Figures and Tables

**Table 1:** Dietary nitrate induced enriched gene ontology terms in old mice. Enriched gene ontology (GO) terms according to biological processes, molecular functions and cellular components. GO terms are ordered by Fisher exact test (i.e., ease score) with highest enriched term at the top of the list. FDR, false discovery rate.

Term	Count	%	*P* value	FDR
Biological process				
Brown fat cell differentiation	15	0.7	1.3*E* − 5	2.4*E* − 2
Rhythmic process	19	0.9	1.4*E* − 3	2.4*E*0
Extracellular structure organization	30	1.5	2.1*E* − 3	3.7*E*0
Negative regulation of lipid metabolic process	6	0.3	2.3*E* − 3	4.1*E*0
Signal transduction	209	10.2	4.0*E* − 3	7.0*E*0
Response to hormone stimulus	29	1.4	4.2*E* − 3	7.3*E*0
Fat cell differentiation	17	0.8	6.1*E* − 3	1.1*E*1
Regulation of cell proliferation	77	3.7	6.9*E* − 3	1.2*E*1
Negative regulation of multicellular organismal process	19	0.9	9.7*E* − 3	1.6*E*1
System development	226	11.0	1.3*E* − 2	2.1*E*1
Response to endogenous stimulus	29	1.4	1.3*E* − 2	2.1*E*1
Extracellular matrix organization	20	1.0	1.4*E* − 2	2.3*E*1
Response to hypoxia	15	0.7	1.7*E* − 2	2.7*E*1

Cellular compartment				
Membrane	703	34.2	6.1*E* − 6	8.8*E* − 3
Integral to membrane	492	24.0	9.8*E* − 6	1.4*E* − 2
Extracellular region part	98	4.8	1.0*E* − 5	1.5*E* − 2
Extracellular region	169	8.2	1.8*E* − 5	2.6*E* − 2
Membrane part	634	30.9	1.9*E* − 5	2.8*E* − 2
Intrinsic to membrane	507	24.7	3.6*E* − 5	5.2*E* − 2
Extracellular matrix	50	2.4	9.3*E* − 5	1.3*E* − 1
Proteinaceous extracellular matrix	48	2.3	1.0*E* − 4	1.5*E* − 1
Extracellular matrix part	22	1.1	3.9*E* − 4	5.6*E* − 1
Basement membrane	18	0.9	1.4*E* − 3	2.0*E*0
Mitochondrial inner membrane	56	2.7	2.0*E* − 3	2.8*E*0
Plasma membrane	291	14.2	4.8*E* − 3	6.6*E*0
Mitochondrion	190	9.3	6.1*E* − 3	8.4*E*0

Molecular function				
Antigen binding	14	0.7	7.1*E* − 5	1.1*E* − 1
Transmembrane receptor activity	88	4.3	1.6*E* − 3	2.6*E*0
Molecular transducer activity	172	8.4	6.1*E* − 3	9.3*E*0
Signal transducer activity	172	8.4	6.1*E* − 3	9.3*E*0
Protein tyrosine kinase activity	28	1.4	9.9*E* − 3	1.5*E*1
cAMP-dependent protein kinase regulator activity	5	0.2	1.0*E* − 2	1.5*E*1
Receptor activity	142	6.9	1.1*E* − 2	1.6*E*1
Oxidoreductase activity, acting on the aldehyde or oxo group of donors, NAD or NADP as acceptor	9	0.4	1.5*E* − 2	2.2*E*1
Kinase regulator activity	16	0.8	1.9*E* − 2	2.7*E*1
Cation transmembrane transporter activity	57	2.8	2.0*E* − 2	2.8*E*1
Copper ion binding	13	0.6	2.1*E* − 2	2.9*E*1
Oxidoreductase activity	10	0.5	2.6*E* − 2	3.4*E*1
Potassium ion binding	16	0.8	2.9*E* − 2	3.8*E*1

**Table 2:** Differentially expressed genes related to calcium pathway in old versus old nitrate treated mice.

Gene symbol	Description	Regulation	Fold change	*P* value
Atp2a1	ATPase, Ca++ transporting, cardiac muscle, fast twitch 1	Up	1.2	0.010
Atp2b2	ATPase, Ca++ transporting, plasma membrane 2	Up	1.3	0.018
Ptk2b	PTK2 protein tyrosine kinase 2 beta	Down	1.2	0.008
Adcy7	Adenylate cyclase 7	Up	1.4	0.001
Adra1a	Adrenergic receptor, alpha 1a	Down	1.5	0.002
Adrb3	Adrenergic receptor, beta 3	Down	1.9	0.004
Cacna1d	Calcium channel, voltage-dependent, L type, alpha 1D subunit	Up	1.4	0.013
Ppapdc2	Calcium channel, voltage-dependent, L type, alpha 1S subunit	Down	1.3	0.010
Camk4	Calcium/calmodulin-dependent protein kinase IV	Up	1.4	0.009
F2r	Coagulation factor II (thrombin) receptor	Up	1.3	0.001
Ednrb	Endothelin receptor type B	Down	1.2	0.045
Gna14	Guanine nucleotide binding protein, alpha 14	Up	1.6	0.031
Itpr2	Inositol 1,4,5-triphosphate receptor 2	Down	1.3	0.001
Itpr3	Inositol 1,4,5-triphosphate receptor 3	Up	1.2	0.024
Itpka	Inositol 1,4,5-trisphosphate 3-kinase A	Down	1.4	0.017
Nos2	Nitric oxide synthase 2, inducible	Down	1.2	0.019
Oxtr	Oxytocin receptor	Down	1.4	0.042
Plcb4	Phospholipase C, beta 4	Up	1.4	0.005
Plcd1	Phospholipase C, delta 1	Up	1.2	0.001
Phka2	Phosphorylase kinase alpha 2	Down	1.2	0.037
Pdgfrb	Platelet derived growth factor receptor, beta polypeptide	Up	1.2	0.007
Calm2	Calmodulin 2	Up	1.2	0.020
Ptger1	Prostaglandin E receptor 1 (subtype EP1)	Up	1.2	0.023
Ptger3	Prostaglandin E receptor 3 (subtype EP3)	Down	1.9	0.020
Prkx	Protein kinase, X-linked	Down	1.2	0.031
Ppp3cc	Protein phosphatase 3, catalytic subunit, gamma isoform	Down	1.3	0.025
P2rx3	Purinergic receptor P2X, ligand-gated ion channel, 3	Up	1.3	0.035
Ryr2	Ryanodine receptor 2, cardiac	Up	1.4	0.007
Camk2g	Similar to Calcium/calmodulin-dependent protein kinase type II gamma chain	Up	1.3	0.010
Sphk2	Sphingosine kinase 2	Down	1.2	0.021

**Table 3:** Enriched genes associated with the biological process of detoxification, antioxidation, and free radical removal, according to Panther Gene Ontology in old versus old nitrate treated mice.

Gene symbol	Description	Regulation	Fold change	*P* value
pAbcc1	ATP-binding cassette, subfamily C (CFTR/MRP), member 1	Up	1.2	0.007
Asna1	arsA arsenite transporter, ATP-binding	Down	1.2	0.025
Ces1d	Carboxyl esterase 3	Down	1.4	0.002
Ces1f	Expressed sequence AU018778	Down	1.6	0.010
Ephx1	Epoxide hydrolase 1, microsomal	Up	1.3	0.015
Gpx3	Glutathione peroxidase 3	Down	1.5	0.001
Gpx4	Heterogeneous nuclear ribonucleoprotein L-like; glutathione peroxidase 4	Down	1.4	0.003
Gsta3	Glutathione S-transferase, alpha 3	Down	2.1	0.039
Gsta4	Glutathione S-transferase, alpha 4	Down	1.5	0.010
Gstm4	Glutathione S-transferase, mu 4	Down	1.3	0.009
Gstt3	Glutathione S-transferase, theta 3	Up	1.2	0.020
Gstz1	Glutathione transferase zeta 1	Down	1.3	0.034
Haghl	Hydroxyacyl glutathione hydrolase-like	Up	1.2	0.006
Mgst1	Microsomal glutathione S-transferase 1	Down	1.3	0.044
Mgst2	Microsomal glutathione S-transferase 2	Down	1.7	0.015
Mgst3	Microsomal glutathione S-transferase 3	Down	1.2	0.044
Mpv17l	Mpv17 transgene, kidney disease mutant-like	Up	1.3	0.001
Nos2	Nitric oxide synthase 2, inducible	Down	1.2	0.019
Pnkd	Paroxysmal nonkinesiogenic dyskinesia	Down	1.4	0.030
Pon3	Paraoxonase 3	Down	1.2	0.002
Prdx4	Peroxiredoxin 4	Up	1.2	0.001
Prdx5	Peroxiredoxin 5	Down	1.2	0.011
Pxmp2	Peroxisomal membrane protein 2	Down	1.2	0.011
Sod1	Superoxide dismutase 1, soluble	Down	1.2	0.015
Sod3	Superoxide dismutase 3, extracellular	Up	1.2	0.015
Tst	Thiosulfate sulfotransferase, mitochondrial	Down	1.3	0.037
Txndc16	Thioredoxin domain containing 16	Up	1.2	0.009
Txnl4a	Thioredoxin-like 4A	Down	1.2	0.002
